# Hospital Meetings

**Published:** 1903-06-20

**Authors:** 


### HOSPITAL MEETINGS.

### THE LIVINGSTONE COLLEGE.

The Commemoration Day proceedings at Livingstone College took place at Leyton, on Wednesday, June 10th. The Bishop of St. Albans presided, and there were present Sir T. Fowell Buxton, Mr. T. F. Buxton, Mr. R. L. Barclay (Treasurer of the College), Mrs. Bishop, Rev. J. T. Inskip (Vicar of Leyton), and a number of medical men, clergy, and ministers of various denominations.

The Principal made a statement concerning the progress of the College.

The Bishop of St. Albans, in the course of an interesting address, said there were many advantages attached to the work of the College, one special one being the help and assistance it gave the missionaries in the preservation of their own health and strength in the work to which they were called. That was a feature of the College which deserved, he ventured to think, special attention. There could be little doubt that the average life of a man abroad was considerably extended when due care was taken to observe the rules of health. He had himself noticed that when in India some years ago, finding that the young soldiers who came out there and led reckless and careless lives, died very soon whilst others who came out there and took ordinary care of themselves lived much longer, he had spoken to the army medical officers often on the subject, and they had told him that if the young soldier would only take real care of himself he might live in India almost as well as anywhere else. What applied to the young soldier applied also to every other Englishman living in the tropics. If the missionaries when they went out to their work abroad were possessed of an elementary knowledge of medicine, and some knowledge of surgery such as could be obtained from a year's course at Livingstone College, he was perfectly certain that every missionary society would feel the benefit of it. He pointed out that a missionary who had a training at that college, even although he was not going to undertake definite medical missionary work and had not taken his diploma, must receive the greatest possible advantage and help from the training received in that college. The natives in India and elsewhere, whilst they might suspect their teaching, had a profound belief in English medicine, and anyone with the most elementary knowledge of medicine and surgery could approach them and influence them in a way in which no one else could. Passing on to speak of definite medical mission work, the Bishop said he was quite convinced that there was no department of mission work in which such great progress had been made as in the medical work.

At the close of the meeting opportunity was given for inspecting the college, and much admiration was expressed with the arrangements.

### LONDON HOSPITAL.

The Quarterly Court of Governors of this hospital was held on June 3, Mr. Charles Cotes, member of the House Committee, being in the chair.

The Secretary, Mr. E. W. Morris, then read the report of the House Committee, which stated that the quinquennial appeal had so far met with a most satisfactory response, and had far exceeded the expectations of the committee. The total amount received either in cash or promises was £134,550. On March 25 the Chairman had received from her Majesty the Queen bank notes for £1,000. This generous and kind act would always be gratefully remembered by the hospital. They were also much indebted to the Lord Mayor, who, in addition to instituting an appeal among City firms, had given a dinner at the Mansion House at his own cost in aid of the funds of the hospital, and had also contributed the sum of £1,000. In connection with the appeal a committee of ladies, with the Countess of Derby at their head, had organised a grand ball at the Albert Hall, for which 3,000 tickets had been sold, so that the committee had doubled the price. The rebuilding of the east wing was proceeding, an anonymous donor having offered to tile the Talbot Ward. The laundry was almost finished, arrangements having been made for the supply of machinery and fittings. Work had also been begun in the west wing, and the basement and half the first floor were in the hands of the builders. Thanks were due to the architect, whose subscription list had reached a total of close on £3,000, which included a donation of £1,000 from himself. Objections had been raised on behalf of the hospital before the assessment committee against the rating of the out-patients' department, but they were informed that they had no grounds for objection. The committee had found it necessary to make three new appointments to the staff—two men had been added as emergency officers and one in charge of special departments. The convalescent home was doing good work. The total of in-patients treated in the last quarter was 2,985, and of out-patients 21,636. Referring to the visit of the King and Queen on June 11 to open the new out-patients' department the committee earnestly hoped that the debt of £55,000 would be cleared off on that occasion.

A vote of thanks to the chairman terminated the proceedings.

